# Draft Genome Sequences of Four Different Strains Belonging to Leptospira interrogans Serovar Pomona Isolated from Mammals in the Island of Sardinia, Italy

**DOI:** 10.1128/MRA.00698-21

**Published:** 2021-09-23

**Authors:** Ivana Piredda, Fabio Scarpa, Daria Sanna, Marco Casu, Maria Nicoletta Ponti, Valentina Chisu

**Affiliations:** a Istituto Zooprofilattico Sperimentale of Sardinia, Animal Health Department, Sassari, Italy; b Department of Veterinary Medicine, University of Sassari, Sassari, Italy; c Department of Biomedical Sciences, University of Sassari, Sassari, Italy; Indiana University, Bloomington

## Abstract

Leptospirosis is a zoonotic disease of global importance caused by a pathogenic group of bacteria belonging to the genus *Leptospira*. Here, we report four draft genome sequences of Leptospira interrogans serovar Pomona isolated in Sardinia (Italy) from four different species of mammals (i.e., dolphin, wild boar, bovine, and fox).

## ANNOUNCEMENT

Leptospirosis is a common and globally diffused zoonosis caused by a pathogenic spirochete belonging to the genus *Leptospira* (1). Exposure to soil or water contaminated with the urine of reservoir animals (mostly rodents) infected with pathogenic species of *Leptospira* is the most common way in which humans or animals contract leptospirosis.

Here, we report four draft genome sequences of Leptospira interrogans isolated in Sardinia (Italy) from the kidneys of four different species of mammals (i.e., dolphin, wild boar, bovine, and fox). The dead animals were necropsied, and kidneys were collected under sterile conditions from each animal. Ethics review and approval were waived because the analyzed animals were found dead. Isolation was performed with 25 mg of tissue using the commercial semisolid Ellinghausen-McCullough-Johnson-Harris (EMJH) medium with EMJH enrichment (Difco, BD, Franklin Lakes, NJ, USA), supplemented with 5-fluorouracil (5-FU; 2 g/liter). After homogenization, the kidney samples were mixed directly into the semisolid medium and incubated aerobically at 29°C for 30 to 40 days. The axenic status was verified using rabbit antiserum. Genomic DNA was extracted from a single colony using the DNeasy blood and tissue kit (Qiagen, Hilden, Germany) according to the manufacturer’s instructions. The genomes were sequenced using whole-genome shotgun (WGS) sequencing performed on the Illumina MiSeq platform by the external core service BMR Genomics (Padua, Italy). The DNA was processed using the Nextera XT DNA library preparation kit (Illumina) following the manufacturer’s instructions. The final library was sequenced on an Illumina MiSeq device using the MiSeq reagent kit v3-600 in 300-bp paired-end runs. The resulting FASTQ files were tested for quality using the FastQC tool implemented in the command line package FASTX-Toolkit v0.7 (2), which was also used for removing adapter sequences (command line: fastx_clipper -e 0.1 -a CTGTCTCTTATACACATCT -l 15) and trimming too-short and low-quality sequences (command line: fastq_quality_filter -v -m 30 -Q 33 -q 25 -p 50). Following the workflow of Scarpa et al. (3), *de novo* assembly was performed using SPAdes v3.13.0 (4) (command line: spades.py --threads 8 -o . --dataset spades_yaml_file.txt - m 128). The coverage for all sequenced genomes was estimated to be ≃100×.

The systematic rank of the annotated genome sequences, verified using PATRIC, the Bacterial Bioinformatics Resource Center (https://www.patricbrc.org/) (5), is as follows: cellular organisms, *Bacteria*, *Spirochaetes*, *Spirochaetales*, *Leptospiraceae*, *Leptospira*, Leptospira interrogans.

Molecular typing of the genomes was performed using multilocus sequence typing (MLST) analysis in PubMLST (https://pubmlst.org/bigsdb?db=pubmlst_leptospira_isolates). The obtained profile (scheme, MLST1=140, MLST2=52, MLST3=58) is associated with genomes belonging to serovar Pomona, serogroup Pomona.

The genomes were very similar to each other. The FASTQ files for the first one (isolate s145, isolated from dolphin kidney) contained 1,081,845 reads with 218,144,564 nucleotides (nt) and 1,063,441 reads with 214,656,053 nt for R1 (read 1) and R2 (read 2), respectively. The assembled file contains 295 contigs, with an estimated genome sequence length of 4,538,424 nt. The average G+C content is 34.98%. The *N*_50_ length is 29,894 bp, while the *L*_50_ count is 52 contigs. The *N*_75_ and *L*_75_ values are 19,228 bp and 101 contigs, respectively. Details on the other three whole genomes are reported in Table 1. All whole draft genome sequences were annotated through the NCBI Prokaryotic Genome Annotation Pipeline (PGAP) (6), using the genetic code 11, by means of the method “Best-placed reference protein set, GeneMarkS-2+.” Information about PGAP can be found at https://www.ncbi.nlm.nih.gov/genome/annotation_prok/. The annotated genome sequence contains 3,781 predicted protein-coding genes (CDS), 37 tRNAs, and 11 rRNA genes.

**TABLE 1:** Details on the whole genomes of Leptospira interrogans

Characteristic	Data for isolate:			
	s145	s146	s147	s180
Host	Dolphin	Wild boar	Bovine	Fox
No. of reads (FastQ R1)	1,081,845	1,316,789	1,243,477	1,394,682
No. of nucleotides (FastQ R1)	218,144,564	268,493,983	255,366,915	274,552,936
No. of reads (FastQ R2)	1,063,441	1,290,137	1,218,543	1,360,913
No. of nucleotides (FastQ R2)	214,656,053	263,207,085	250,011,348	268,589,532
No. of contigs	295	246	311	292
GC content (%)	34.98	34.96	34.98	34.98
Contig *L*_50_	52	35	38	46
Contig *N*_50_ (nt)	29,894	41,803	38,610	32,915
Contig *L*_75_	101	69	79	92
Contig *N*_75_ (nt)	19,228	23,082	20,675	18,945
Genome length (nt)	4,538,424	4,554,959	4,613,019	4,537,813
No. of predicted CDS	3,781	3,785	3,845	3,457
No. of tRNAs	37	37	37	37
No. of rRNAs	11	7	13	8

### Data availability.

The reported whole-genome shotgun sequences of Leptospira interrogans strains have been deposited in NCBI GenBank under the following accession numbers: JAHERX000000000 (JAHERX010000001 through JAHERX010000275), JAHERY000000000 (JAHERY010000001 through JAHERY010000237), JAHERZ000000000 (JAHERZ010000001 through JAHERZ010000246), and JAHESA000000000 (JAHESA010000001 through JAHESA010000262). They can also be found under BioProject accession number PRJNA731636 and BioSample accession numbers SAMN19296173, SAMN19296548, SAMN19317300, and SAMN19317364. The version described in this paper, belonging to isolate s145 (dolphin), is JAHERX010000000.
